# Editorial: Updates on hidradenitis suppurativa – Pathogenesis, diagnosis and treatment

**DOI:** 10.3389/fmed.2023.1209545

**Published:** 2023-05-10

**Authors:** Emanuele Scala, Alexander Zink

**Affiliations:** ^1^Department of Dermatology and Venereology, Medical Center - University of Freiburg, Faculty of Medicine, University of Freiburg, Freiburg, Germany; ^2^Division of Dermatology and Venereology, Department of Medicine Solna and Center for Molecular Medicine, Karolinska Institutet, Stockholm, Sweden; ^3^Department of Dermatology and Allergy, School of Medicine, Technical University of Munich, Munich, Germany; ^4^Division of Dermatology and Venereology, Department of Medicine Solna, Karolinska Institutet, Stockholm, Sweden

**Keywords:** hidradenitis suppurativa, acne inversa, pathogenesis, diagnosis, treatment

Hidradenitis suppurativa (HS) is a chronic, debilitating, inflammatory disease of the pilosebaceous unit characterized by painful nodules, abscesses, sinus tracts, and scar formation, affecting ~1% of the general population with common onset in young adulthood (1). HS is clinically under-recognized, and the diagnosis takes, in median, 7 years to be made (2). It has an extremely high comorbidity burden, rate of hospitalization, and recalcitrance to treatment deeply affecting patients' quality of life (1, 2). To date, the pathogenesis of HS is only partially understood and the lack of knowledge regarding the intersection of genetics, immunology and environmental risk factors (e.g., smoking, obesity, and mechanical friction) is a major obstacle to improving treatment for patients with HS (1–4).

In this Research Topic, we invited scientists to summarize the latest updates on HS pathogenesis, diagnosis, and treatments.

The topic started with a non-interventional, cross sectional monocentric study by Sabat el al. revealing a number of sex-related differences for disease risk factors, clinical aspects, and comorbidity in 500 patients suffering from HS (303 women and 197 men). Central obesity was more frequent in women while extensive cigarette smoking and acne vulgaris were more commonly found among male patients. The latter reported more often lower blood HDL-cholesterol levels, higher blood glucose levels, and experienced arterial hypertension than HS afflicted women. Clinically, the axillary skin sites were affected significantly more frequently in male than in female HS patients. Conversely, the groin was involved more frequently in women than in male HS patients. Moreover, women showed a higher number of skin sites with inflammatory nodules, whereas fistulas were observed more frequently in men. Although the treatment of fistulas or inflammatory nodules should be different, in this case with more excisions in men and more conservative treatments in women, no significant differences in the therapeutic procedure between the two groups were observed, demonstrating that the HS therapy algorithms are still being under development. Thus, sex-specific differences should be also taken into account in the prevention as well as medical and surgical treatment of HS patients. Furthermore, a frequent monitoring of clinical parameters (e.g., blood pressure) and clinical chemistry (e.g., HDL-cholesterol) should be an indestructible part of the medical consultation for both sexes.

The topic proceeds with an opinion article by Luporini et al.. The authors approach the question of the therapeutic intervention from a different angle considering HS as extraintestinal manifestations (EIM) and therefore it might be treated as such. The theory was explored on three main findings: (*i*) the genetic predispositions factors associated with increased risk of HS in inflammatory bowel disease (IBD) (SULT1B1 and SULT1E1), (*ii*) the same environmental factor predisposition with increased risk of shooting both HS and CD (i.e., smoking), and (*iii*) the good response to treatment with anti-TNF-α in HS (as well as in dermatological EIM).

Dermatologists are often faced with treating a severe form of the disease that does not respond to conventional therapies (e.g., immunosuppressants, topical and systemic antibiotics), and patients also know that none of these agents work well-enough (1, 5). Therefore, the management of HS patients is difficult and substantially increases health care resource utilization and costs (5). In this context, Dell'Antonia et al. propose cryotherapy as a useful and effective adjunctive treatment of persistent HS nodules of the axilla, groin, and gluteal region, not responding to at least 16 weeks of systemic medical therapy (e.g., antibiotics, estro-progestinic pills, and adalimumab). This retrospective observational study involves 23 HS patients (17 female and 6 male subjects) showing a complete resolution, clinically and ecographically documented, in 88.7% of the lesions treated after one cryotherapy treatment with no recurrence of the regressed nodules after 6 months. The beauty of this procedure is that can be easily performed by any dermatologist and does not require great experience. Moreover, cryotherapy is less expensive than other procedures, and it is usually available during the visit in a common outpatient setting; thus, it can be a valid alternative to the local surgery.

Finally, Daoud et al. provide a comprehensive review of the various scores used to evaluate the severity and response to treatment of HS. Since HS can be difficult to score accurately due to its clinical heterogeneity, and there are currently numerous scoring systems in use, the authors' aim was to compare the various scores used for individual patients and to provide a detailed narrative review of the scores used to date. This review included 19 scores, and the authors illustrate that for some patients, the scores do not consistently correlate with each other, either in evaluating the severity at a time-point or in evaluating the response to treatment. Some patients may be considered responders according to some scores, but non-responders according to others, which highlights the clinical heterogeneity of the disease. The article emphasizes that the choice of a score can lead to different interpretations of the response to treatment, and potentially change the results of a randomized clinical trial. This has significant implications for clinicians and researchers who need to select the most appropriate scoring system to accurately evaluate the severity and response to treatment of HS. Overall, the article provides an insightful review of the current scoring systems used to evaluate HS, and highlights the need for a standardized scoring system to ensure consistency and accuracy in evaluating the severity and response to treatment of this challenging disease. The authors' work will be valuable to clinicians and researchers in this field.

In conclusion, this collection proves that HS is a complex disease with multifactorial etiologies. Therefore, additional efforts are required from scientists and clinicians to understand the molecular mechanisms underlying the disease, the extra-cutaneous manifestations and comorbidity. This may result in finding new ways of treating or curing this debilitating skin disease.

## Author contributions

All authors listed have made a substantial, direct, and intellectual contribution to the work and approved it for publication.

## References

[1] Scala E Cacciapuoti S Garzorz-Stark N Megna M Marasca C Seiringer P. Hidradenitis suppurativa: where we are and where we are going. Cells. (2021) 10:2094. 10.3390/cells10082094 34440863 PMC8392140

[2] Vellaichamy G Amin AT Dimitrion P Hamzavi Z Zhou L Adrianto I. Recent advances in hidradenitis suppurativa: role of race, genetics, and immunology. Front Genet. (2022) 13:918858. 10.3389/fgene.2022.918858 36092908 PMC9458948

[3] Scala E Di Caprio R Cacciapuoti S Caiazzo G Fusco A Tortorella E. A new T helper 17 cytokine in hidradenitis suppurativa: antimicrobial and proinflammatory role of interleukin-26. Br J Dermatol. (2019) 181:1038–45. 10.1111/bjd.17854 30829398

[4] Scala E Balato A Marasca C Di Caprio R Raimondo A Cacciapuoti S. New insights into mechanism of Notch signaling in hidradenitis suppurativa. G Ital Dermatol Venereol. (2020) 155:529–30. 10.23736/S0392-0488.18.06083-2 29998713

[5] Marasca C Tranchini P Marino V Annunziata MC Napolitano M Fattore D. The pharmacology of antibiotic therapy in hidradenitis suppurativa. Expert Rev Clin Pharmacol. (2020) 13:521–30. 10.1080/17512433.2020.1762571 32364806

